# Detection of Bacteria Bearing Resistant Biofilm Forms, by Using the Universal and Specific PCR is Still Unhelpful in the Diagnosis of Periprosthetic Joint Infections

**DOI:** 10.3389/fmed.2014.00030

**Published:** 2014-09-16

**Authors:** Batool H. Zegaer, Anastasios Ioannidis, George C. Babis, Vassiliki Ioannidou, Athanassios Kossyvakis, Sotiris Bersimis, Joseph Papaparaskevas, Efthimia Petinaki, Paraskevi Pliatsika, Stylianos Chatzipanagiotou

**Affiliations:** ^1^Department of Biopathology and Clinical Microbiology, Aeginition Hospital, Athens Medical School, Athens, Greece; ^2^Department of Nursing, Faculty of Human Movement and Quality of Life Sciences, University of Peloponnese, Sparta, Greece; ^3^2nd Orthopaedic Department, Konstantopouleio General Hospital, University of Athens, Athens, Greece; ^4^National Influenza Reference Laboratory of Southern Greece, Institut Pasteur Hellénique, Athens, Greece; ^5^Department of Statistics and Insurance Science, University of Piraeus, Piraeus, Greece; ^6^Department of Microbiology, Medical School, National and Kapodistrian University of Athens, Athens, Greece; ^7^Department of Microbiology, Medical School, University of Thessaly, Larissa, Greece

**Keywords:** periprosthetic joint infection, culture, PCR, biofilms, antimicrobial resistance

## Abstract

Intraoperative conventional bacteriological cultures were compared with different polymerase chain reaction (PCR) methods in patients with total joint arthroplasties. The isolated bacteria were investigated for biofilm formation, and the biofilm forming strains, in their planktonic and biofilm forms, were further tested for their antimicrobial resistance against several clinically important antimicrobials. Forty four bone and joint samples were included and classified as infected or non-infected according to standard criteria for periprosthetic hip and knee infections. For the bacteriological diagnosis, conventional culture, two types of universal PCR and species specific PCR for three selected pathogens (*Staphylococcus aureus*, *Staphylococcus epidermidis*, and *Pseudomonas aeruginosa*) were applied. Biofilm formation determination was performed by the tissue culture plate method. Antimicrobial susceptibility of the planktonic bacteria was performed by the minimal inhibitory concentration determination and, of the biofilm forms, by the minimal inhibitory concentration for bacterial regrowth from the biofilm. Twenty samples were culture positive, with *S. epidermidis*, *S. aureus*, or *P. aeruginosa*. All PCR methods were very ineffective in detecting only one pathogen. All isolates were biofilm positive and their biofilm forms, were highly resistant. In this study, compared to PCR, culture remains the “gold standard.” The biofilm formation by the causative bacteria and the concomitant manifold increased antimicrobial resistance may explain the clinical failure of treatment in some cases and should be considered in the future for therapeutic planning.

## Introduction

Periprosthetic joint infection (PJI) is one of the most serious complications causing a high grade of morbidity in patients with total joint arthroplasty. Actually, the rate of infection after total hip or knee arthroplasty ranges from below 1 up to 5% and it rises slightly in case of revision procedures (1, 2).

The accurate diagnosis of bone and joint infections, has for long been confounded by the difficulty of retrieval and detection of microorganisms, and is still a challenge to the treating physician (1, 3).

The clinical diagnosis can be assisted by laboratory tests like white cell count and differentiation, erythrocyte sedimentation rate (ESR), C-reactive protein (CRP), and others, but there is no gold standard, having a perfect sensitivity and specificity for diagnosing the infection, apart from identifying the infective bacteria (4).

The distinction between septic and non-septic loosening is difficult in most cases, impeding clinical evaluation (5). The risk of a PJI increases greatly due to the long stay, in the body, of the implant, which can be colonized by microbes from a distant infectious focus through the hematogenous route at any time following implantation (1). Therefore, an increase in the number of PJIs is expected in the coming years.

Currently, periprosthetic infection is frequently diagnosed by isolation of one or more organisms from the periprosthetic tissue or fluid with use of conventional culture techniques, and the culture’s results are usually considered the standard, which are compared with other diagnostic tests (6).

Among the various bacterial virulence factors, a very important one, is the ability to form biofilms (7). In a biofilm, the bacteria are attached to each other and adhering to the artificial materials and medical devices such as contact lenses, artificial heart valves, joint replacements, etc., they produce an extracellular polymeric substance (ESP) consisting of carbohydrate (exopolysaccharide) (8). The production of biofilms protects the bacteria from the effect of the antibiotic action, in addition to the inhibition of the phagocytic cells, and impedes the function of the T- and B-lymphocytes (9–11).

The detection and isolation of causative microorganisms is the first important step for the successful treatment of PJI (12). Many diagnostic methods are applied in order to isolate the causative microorganisms in patients with symptoms of a failed arthroplasty. Some conventional methods, like culture of aspirated joint fluid, can be performed preoperatively. However, the preoperative aspiration does not always precede and the surgeons often do not become suspicious of an infection until the revision arthroplasty operation is underway (4).

Molecular techniques overcome some of the limitations of conventional microbiological diagnostic procedures. More than 10 years have passed since the introduction of molecular methods into the diagnosis of orthopedic infections; these methods are still a matter of research and discussion. Gallo et al. (13) as well as Spangehl et al. (14) investigated how the polymerase chain reaction (PCR) might play an essential role in PJI diagnosis. They pointed out the advantages of accuracy and speed, as well as the possible disadvantages, like the false-positive results, misinterpretation, and the expensive equipment requirements (13, 14).

Despite the numerous studies already published, the role of molecular techniques, like PCR, in the diagnosis of PJI, still remains vague and unclear. Therefore, the effectiveness of these techniques has not yet been verified in the routine of the clinical microbiology laboratory diagnosis for PJI (5).

The aim of the present study is to compare the conventional cultures with two PCR methods (universal and specific), for the fast and accurate diagnosis of PJI in 44 patients who had a primary or failed total joint arthroplasty. In addition, the isolated bacteria were investigated with respect to biofilm formation. The biofilm forming strains, in their planktonic and biofilm forms, were further tested for their antimicrobial resistance against several clinically important antimicrobials, used for the treatment of PJI.

## Materials and Methods

### Patients

The study included 44 bone and joint samples collected intraoperatively from an equal number of patients. Thirteen from patients with a preoperative diagnosis of infected, and 31 from patients with a preoperative diagnosis of non-infected be based on a clinician’s independent medical judgment (depending on the history of the patient, the clinical examination, such as constant pain, warmth and effusion, erythema, delayed healing of the wound, plain x-ray, and other patient individualized criteria). All the patients were treated in the 1st Department of Orthopedic Surgery “ATTIKON” University Hospital of the Athens Medical School. The age of the patients ranged from 31 to 85 years. Patients were classified in two groups, with respect to the final clinical diagnosis, as infected or non-infected cases.

The clinical determination of deep infection (deep around the artificial implants) was according to the criteria of international standard of PJI (15).

All the patients underwent preoperative general blood examination including white blood cell (WBC) count, ESR, and CRP determination.

### Sample collection and culture

The patient samples used in the study were bone, tissue, or aspiration fluid, which had been taken intraoperatively from the patients with total hip or total knee arthroplasty (more than one sample had been collected from each patient), as routine diagnostic procedure. Bacteriological examination included conventional culture on growth media for aerobic and anaerobic bacteria and direct sample microscopy of Gram stained smears. The identification of the isolated bacteria was performed by conventional bacteriological methods: API-E and API-NE (Biomerieux, Marcy-I’ Etoile/France) for gram-negative bacteria and coagulase and DNAase for *Staphylococcus* spp.

### Biofilm production determination

All isolated bacterial strains were investigated for their ability to form biofilms by the tissue culture plate (TCP) method as described by Christensen et al. (16) and Baldassarri et al. (17), with a modification in duration of incubation, which was extended to 24 h (18).

Briefly, a bacterial suspension was prepared from a blood agar plate culture in trypticase soy broth at opacity of 0.5 McFarland standards and cultured overnight at 37°C. The next day, 100 μl of the overnight culture were added to 200 μl tryptose broth and placed in a micro titer tray well, mixed and incubated overnight at 37°C. The next day, the wells were carefully emptied and washed three times with phosphate buffered saline (PBS). The plate was allowed to dry at 60°C for 1 h and then stained with Hucker’s crystal violet (2 g crystal violet, 20 ml 95% alcohol, 0.8 g ammonium oxalate, and 80 ml distilled water). The excess stain was washed off with distilled water, excess water was removed, and the plates were read with an ELISA reader at 570 nm (19). The cut-off value was calculated as mean ± 2SD of the values of 10 wells processed the same way but without bacteria. Values above the cut-off were considered positive for biofilm formation. Each strain was tested in quadruplicate.

### MIC determination

Antimicrobial susceptibility of the planktonic bacterial forms was performed and interpreted by determination of the minimal inhibitory concentration (MIC) using the standard broth dilution method according to the guidelines of the Clinical Laboratory Standards Institute (20, 21). The antimicrobials included, were those of importance in the clinical practice for treating the isolated bacterial species: ciprofloxacin, moxifloxacin, erythromycin, linezolid, daptomycin, teicoplanin, vancomycin tigecyclin, and cotrimoxazole for *Staphylococcus aureus* and *Staphylococcus epidermidis* and imipenem, meropenem, ceftazidime, aztreonam, tobramycin, ciprofloxacin, amikacin, cefepime, and cotrimoxazole for *Pseudomonas aeruginosa*.

### Minimal inhibitory concentration for bacterial regrowth from the biofilm determination

The strains producing biofilms were further tested for their antimicrobial susceptibility by determination of the minimal inhibitory concentration for bacterial regrowth from the biofilm (MICBR) using a modified broth dilution method as described previously (22).

Serial dilutions of the antimicrobial in Mueller Hinton broth, corresponding to the concentrations used for the MIC determination of the planktonic forms, were prepared and poured into the micro titer plates, which contained the bacterial biofilm and incubated at 35°C for 48 h. The growth of planktonic bacteria was visualized by the development of turbidity in the medium. The MICBR was defined as the lowest concentration showing no growth in the medium as observed by a complete clarity. Each strain was tested in quadruplicate.

The results were assessed using the breakpoints given by the guidelines of the CLSI (20, 21).

### Polymerase chain reaction

Bacterial DNA extraction was performed by means of the protocol of the “Insta Gene Matrix” method (Bio Rad Laboratories, CA, USA) The extracted DNA was stored at −20°C until the time of use.

The types of PCR that were performed for the detection of the causative pathogens were: a) two types of universal PCR (Nr.1 and 2) detecting the 16S rRNA gene by different protocols (23, 24), followed by sequencing of the product for the identification of the species, and b) the species specific PCR, for three selected pathogens: *S. aureus* (25), *S. epidermidis* (26), and *P. aeruginosa* (27). All the PCR primers and annealing temperatures are depicted in Table 1. For all PCR methods, controls were run in parallel with extracted DNA from the following reference strains: *Escherichia coli* ATCC 25922, *P. aeruginosa* ATCC 27853, *S. epidermidis* ATCC 35984, *S. aureus* ATCC 29213, and *Streptococcus pneumoniae* ATCC 49619.

**Table 1:** **PCR primers and annealing temperatures for the detection of bacteria causing periprosthetic joint infections**.

Target gene for	Sequence (5′ → 3′)	Annealing temp. (°C)	Product (bp)
16S rRNA universal 1 (24)	AGAGTTTGATCCTGGCTCAG	59	~1380
	GACGGGCGGTGTGTACAA	
16S rRNA universal 2 (23)	AGTTTGATCCTGGCTCAG	55	~1450
	AGGCCCGGGAACGTATTCAC	
*Staphylococcus aureus* (25)	CTTTGTCGGTACACGATATTCTTCACG	54	108
	CGTAATGAGATTTCAGTAGATAATACAACA	
*Staphylococcus epidermidis* (26)	ATCAAAAAGTTGGCGAACCTTTTCA	50	124
	CAAAAGAGCGTGGAGAAAAGTATCA	
*Pseudomonas aeruginosa* (27)	GGGGGATCTTCGGACCTCA	58	956
	TCCTTAGAGTGCCCACCCG	

Each reaction of PCR consisted 0.4 μM of forward primer, 0.4 μM of reverse primer, 2.5 units/reaction HotStar Taq DNA Polymerase (Qiagen), 1X PCR Buffer provides a final concentration of 1.5 mM MgCl_2_, 200 μM of each deoxynucleotide triphosphates (dNTP), 5 μl DNA sample, and PCR-grade water until they completed 50 μl of reaction volume. The conditions we used are: 1 cycle (94°C for 5 min), 35 cycles (94°C for 1 min, annealing temperature as described in Table 1 for each pair of primers for 1 min, 72°C for 1 min), and 1 cycle (72°C for 10 min), storing at 4°C.

### Statistical analysis

The statistical analysis was performed using the statistical package SPSS for Windows (version 20.0) in order to disclose any significant differences between the percentages of antimicrobial susceptibility of the planktonic and the biofilm bacterial forms. The analysis was done by applying an appropriate hypothesis test concerning the difference between the proportions of two samples. The normal approximation to the binomial distribution was used. Additionally, appropriate parametric and non-parametric tests were used for comparing quantitative variables such as CRP, ESR, and WBC.

## Results

### Sample origin, sample species, culture, and inflammation markers

A total of 44 samples were analyzed in this study: 20 were culture positive, with the following organisms: *S. epidermidis* (15 isolates), *S. aureus* (4 isolates), *P. aeruginosa* (1 isolate), and 24 were culture negative results (Tables 2A,B).

**Table 2:** **The results of universal and specific PCR technique for the culture (A) positive samples and (B) negative samples**.

Patient no.	Preoperative diagnosis	Culture	Universal 1 PCR [19]	Universal 2 PCR [20]	PCR *S. aureus*	PCR *S. epidermidis*	PCR *P. aeruginosa*
**(A)**							
1	Infected	*S. epidermidis*	N	N	N	N	N
2	Infected	*S. epidermidis*	P/no id.	P/no id.	P	P	P
3	Non-infected	*S. aureus*	N	N	N	N	N
4	Infected	*S. aureus*	N	N	N	N	N
5	Infected	*S. epidermidis*	P/no id.	P/no id.	P	P	N
6	Infected	*S. epidermidis*	N	N	N	N	N
7	Non-infected	*S. epidermidis*	N	N	N	N	N
8	Infected	*S. epidermidis*	N	N	N	N	N
9	Non-infected	*S. epidermidis*	N	N	N	N	N
10	Infected	*S. epidermidis*	N	N	N	P	N
11	Infected	*S. aureus*	N	N	P	N	N
12	Infected	*S. aureus*	P/no id.	P/no id.	P	P	P
13	Infected	*P. aeruginosa*	N	N	N	N	P
14	Infected	*S. epidermidis*	N	N	P	N	N
15	Infected	*S. epidermidis*	N	N	N	N	N
16	Non-infected	*S. epidermidis*	N	P/no id.	P	P	P
17	Non-infected	*S. epidermidis*	N	N	N	N	N
18	Non-infected	*S. epidermidis*	N	N	P	N	N
19	Infected	*S. epidermidis*	N	N	P	N	P
20	Non-infected	*S. epidermidis*	N	N	P	P	N
**(B)**							
21	Non-infected	N	N	N	N	N	N
22	Non-infected	N	N	N	N	N	N
23	Non-infected	N	N	N	N	N	N
24	Non-infected	N	N	N	N	N	N
25	Non-infected	N	N	N	N	P	N
26	Non-infected	N	N	N	P	P	N
27	Non-infected	N	N	N	N	N	N
28	Non-infected	N	N	N	N	N	N
29	Non-infected	N	N	N	N	N	N
30	Non-infected	N	N	N	N	P	N
31	Non-infected	N	N	N	P	P	P
32	Non-infected	N	N	N	N	N	N
33	Non-infected	N	N	N	N	N	N
34	Non-infected	N	N	N	N	N	N
35	Non-infected	N	N	N	N	N	N
36	Non-infected	N	N	N	P	P	N
37	Non-infected	N	N	N	N	P	P
38	Non-infected	N	N	N	N	P	N
39	Non-infected	N	N	N	N	N	N
40	Non-infected	N	N	N	N	N	N
41	Non-infected	N	N	N	N	P	N
42	Non-infected	N	N	N	N	N	N
43	Non-infected	N	N	N	N	N	N
44	Non-infected	N	N	N	N	N	N

*N = negative, P = positive, no id. = no identification*.

There was no statistically significant relation, either between culture results and patients’ gender or sample origin (total hip or total knee). However, the sample species proved to be critical for the culture outcome: the tissue and bone samples gave significantly more positive culture results than the aspiration fluid (*p* < 0.001, results not shown).

Sensitivity and specificity of the culture in relation to the presumed preoperative evaluation were 100 and 77.4%, respectively with a Receiver operating characteristic (ROC) area of 0.812–0.962 (ROC value = 0.887).

All three inflammation markers, CRP, ESR, and WBC were significantly higher in patients with a positive culture (*p* < 0.001 for all, Table 3).

**Table 3:** **White blood cell (WBC), C-reactive protein (CRP), and erythrocyte sedimentation rate (ESR) preoperative determination in the blood of the patients subject to total joint arthroplasty**.

Parameter	Mean value (S.D.)			*p*-Value (non-infected vs. infected)
	Total (*N* = 44)	Non-infected (*N* = 31)	Infected (*N* = 13)	
WBC (X 10^3^ cells/μl)	9.33 (2.889)	7.72 (1.294)	13.15 (1.790)	<0.001
CRP (mg/dl)	5.67 (7.609)	1.56 (1.085)	15.47 (7.528)	<0.001
ESR	34.36 (27.510)	20.71 (12.006)	66.92 (26.859)	<0.001

### Polymerase chain reaction

**Figure 1:**
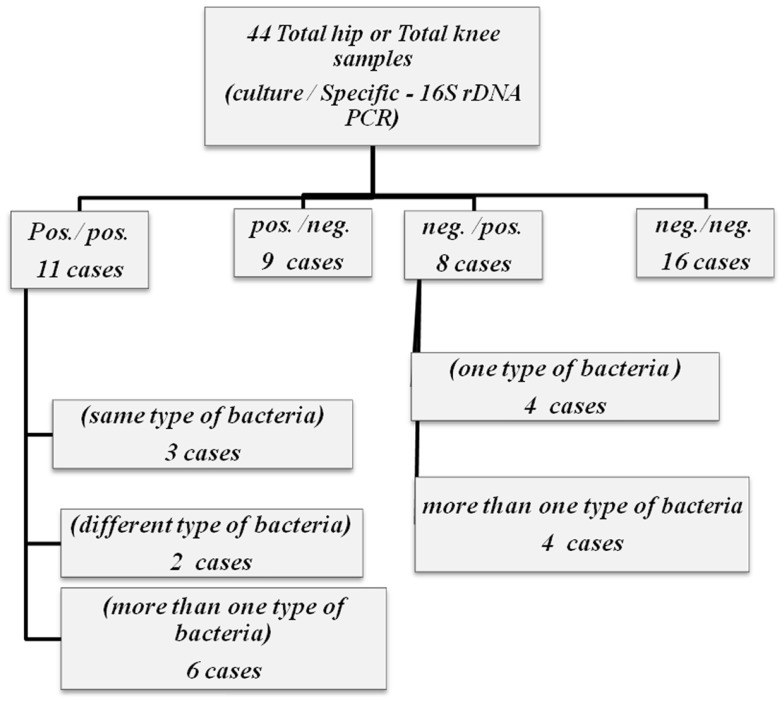
**Flipchart summarizing the results of both culture and specific – 16S rDNA for 44 total hip and total knee samples**.

Both universal PCR methods showed very contradictory results in relation to culture. Although there was an agreement between negative culture and negative PCR for both universal PCR tests, they both failed to detect bacterial 16S rDNA in most culture positive samples. When 16S rDNA was detected, identification on species level through sequencing was impossible; as in all 16S rDNA positive samples, the sequencing reaction was blocked after approximately 60 bp, probably due to the presence of more than one bacterial species (Tables 2A,B). All three specific PCR tests were equally ambiguous, being very ineffective in detecting only one pathogen and giving very discrepant results in relation to culture (Tables 2A,B; Figure 1).

In reference to the preoperative clinical evaluation, sensitivities and specificities of the PCR methods were as follows: universal PCR 1 23.1 and 100% (24), universal PCR 2 23.1 and 96.8% (23), PCR specific for *S. aureus* 46.2 and 80.6%, PCR specific for *S. epidermidis* 30.8 and 67.7%, and *P. aeruginosa* 30.8 and 90.3%.

### Biofilm formation, MIC, and MICBR

All the strains were positive for biofilm production. The great majority of the biofilm forms were resistant to all antimicrobials. In antimicrobial concentrations far higher than the breakpoints, bacterial regrowth from the biofilms was still possible. The results for *S. aureus* and *S. epidermidis* are shown in Table 4. Regarding the MIC (value in microgram per milliliter in parenthesis) of the one isolated strain of *P. aeruginosa*, the strain was susceptible to meropenem ( ≤1), ceftazidime (2), aztreonam (4), tobramycin ( ≤2), ciprofloxacin (0.25), amikacin ( ≤8), and cefepime (2) and intermediate to imipenem (4). The respective MICBR values were at least the fourfold of the MIC values for imipenem, meropenem, ceftazidime, aztreonam, tobramycin, and cefepime, showing significant resistance of the biofilm forms with respect to bacterial regrowth (*p* < 0.001). For ciprofloxacin and amikacin, the MICBR values were identical to the MIC values; thus, these two antibiotics seemed to suppress *in vitro P. aeruginosa* regrowth from the biofilm under corresponding clinical therapeutic dosages.

**Table 4:** **Antimicrobial resistance rates of planktonic and biofilm forms of *S. epidermidis* and *S. aureus* strains isolated from the samples of the patients with periprosthetic joint infection**.

Antimicrobial	*S. epidermidis* (*n* = 15)			*S. aureus* (*n* = 4)		
	Planktonic *n* (%)	Biofilm *n* (%)	*p*-value	Planktonic *n* (%)	Biofilm *n* (%)	*p*-value
Ciprofloxacin	5 (33.33)	11 (73.33)	<0.001	2 (50)	3 (75)	<0.001
Moxifloxacin	9 (60)	12 (80)	<0.001	2 (50)	3 (75)	<0.001
Erythromycin	11 (73.33)	15 (100)	<0.001	2 (50)	4 (100)	<0.001
Linezolid	0 (0)	13 (86.66)	<0.001	0 (0)	4 (100)	<0.001
Daptomycin	1 (6.66)	15 (100)	<0.001	0 (0)	4 (100)	<0.001
Teicoplanin	1 (6.66)	15 (100)	<0.001	0 (0)	4 (100)	<0.001
Vancomycin	0 (0)	12 (80)	<0.001	0 (0)	3 (75)	<0.001
Tigecycline	6 (40)	10 (66.66)	<0.001	1 (25)	4 (100)	<0.001
Cotrimoxazole	7 (46.66)	14 (93.33)	<0.001	1 (25)	4 (100)	<0.001

## Discussion

The diagnosis of the prosthetic joint infection includes a set of laboratory tests. In the present study, like in previous reports (6, 28, 29), the traditional first line laboratory tests including the inflammation markers CRP, ESR, and WBC proved to have a very good sensitivity, when correlated with the clinical evaluation (Table 3).

Culture is still the gold standard for diagnosis of prosthetic joint infection, offering the possibility of the antimicrobial susceptibility testing, but it is time-consuming in identifying the causative microorganisms. However, the drawbacks of the cultural procedures are the limited sensitivity, in addition to the false negative results in patients receiving antimicrobials (6, 30). Our study comprised 44 cases, 20 cultures-positive, and 24 negative (more than one sample had been taken from each patient). In seven cases with a presumed preoperative absence of infection (non-infected), culture was positive, which may have been through sample contamination during handling (collection, transport, and processing).

In a prospective study involving revision of arthroplasty in 297 patients with a total of 41 infections, Atkins et al. (31) pointed out that only 65% of all samples collected from the infected joints were culture positive. They recommended collecting five or six culture samples from each patient and suggested that the accurate diagnosis of infection should be considered as the growth of the identical microorganism on culture in three or more samples (6). Practically, this procedure is difficult, but at the same time, it could increase the possibility of detecting the causative microorganism.

All isolated bacterial strains were positive for biofilm formation *in vitro*, by the TCP performed as previously described (16, 17). The *in vivo* biofilm synthesis gives bacteria the ability to cause infection and impedes their cultural isolation from the samples because the biofilms adhere strongly to the colonized biological surface. The use of ultrasound to expel bacterial cells from the biofilms adherent to the surface of removed implants (sonication) increases the effectiveness and the sensitivity of microbiological studies to determine the underlying microorganisms, but, at the same time, increases the risk of contamination through the more complicated sample handling (32, 33).

The antimicrobial resistance rates of the planktonic and the biofilm forms of the isolated bacteria, tested by the MIC and MICBR determination, showed increased antimicrobial resistance of the biofilm forms to the vast majority of the antimicrobials, with MICs far above those of the planktonic forms and above the breakpoints corresponding to the therapeutic clinical implementation (Table 4). This may explain the failure of treatment in some cases, where despite the antimicrobial susceptibility to a certain antimicrobial *in vitro*, the infection still exists after the appropriate treatment (clinical resistance) (34–37). Although there are no standard procedures for the determination of MICBR, our results are in agreement with previously reported data, with respect to the role of biofilms in the increase of the bacterial antimicrobial resistance (38). Furthermore, they confirm that the experimental conditions used, led to biofilm formation. Biofilm production in *S. epidermidis* and *P. aeruginosa* belongs to the most significant virulence factors for the expression of pathogenicity in infectious diseases (16, 38, 39).

The most important part in this study was the application and evaluation of PCR. Based on the literature, PCR can be considered one of the very helpful diagnostic tools, used in cases of arthritis, especially when culture is negative. Fenollar et al. (3) reported remarkable results for cloning and sequencing of 16S rDNA amplicons. They found a perfect compatibility between culture and PCR results in 475 of 525 samples (90.5%). Kordelle et al. (40) sequenced the 16S rDNA amplicons and found 100% agreement between PCR and culture, but the study included only seven cases of PJI. In the present study, the universal 16S rDNA PCR technique was totally unhelpful for the bacterial identification. Although it detected bacterial DNA in many cases, with a significant agreement with culture (*p*-value 0.031), there was no identification of the DNA product after sequencing. This problem might be due to the presence of more than one bacterial species (including the causative microorganism), caused through sample handling and processing. To be effectual, the universal PCR requires specimens containing a single bacterial strain; otherwise, the identification after sequencing is impossible.

The specific PCR showed better results but still very discrepant compared to culture, the *p*-values were 0.014, 0.583, and 0.226 for PCR *S. aureus*, *S. epidermidis*, and *P. aeruginosa*, respectively in comparison with culture. Published results from different studies on the use of PCR to detect prosthetic joint infection have made PCR a technique not yet widely accepted in routine examination. The sensitivity and specificity of broad-range PCR from synovial fluid and/or tissue for the diagnosis of prosthetic joint infection have been reported to be between 50–92% and 65–94%, respectively (41–44). In the present report, sensitivity and specificity of all the PCR methods applied were very diverse and in none of the cases did they fulfill the criteria of a reliable diagnostic method for the bacteriological diagnosis of PJI (45).

In the present report, the molecular methods proved to be inappropriate for a reliable bacterial diagnosis, the culture remaining still “the gold standard.” When compared to conventional microbiological procedures, PCR analysis is still hindered by higher costs, false-positive results, and interpretative problems. Currently, under these circumstances it is not justifiable to introduce molecular methods into the schemes used to diagnose prosthetic joint infection (13).

The detection and identification of bacterial RNA, rather than DNA, can be a new approach to the laboratory diagnosis of prosthetic joint infection, by reverse transcription. RNA is present only in viable bacteria and, thus, it could be more reliable in disclosing active infections. On the other hand, the much shorter half-life of RNA would make its role as a contaminant less likely (46). Rasouli et al. have been evaluating the new approach of multiplex PCR (Ibis T5000 universal Biosensor), which depend on pan-genomic amplification, and mass spectrometry for culture negative cases in patients with suspicion of PJI and the results were promising (15).

In conclusion, our results showed that the molecular diagnostic methods, like PCR, did not increase the detection rate of prosthetic joint infection, compared to culture. Improved PCR methods may be considered in the near future and play an important role in the diagnosis of bone infections as a complement to culture, in cases where a small amount of samples are available for examination, or when culture is negative after 24 h of incubation for patients with suspected prosthetic joint infection, as well as in the diagnosis of samples taken from patients receiving antimicrobial therapy.

## Conflict of Interest Statement

The authors declare that the research was conducted in the absence of any commercial or financial relationships that could be construed as a potential conflict of interest.

## Abbreviations

AAOS, American Academy of Orthopaedic Surgeons; APIE, APINE, microorganism identification kits for gram-negative bacteria (biomerieux API, 69280 Marcy-I’ Etoile/France); CLSI, Clinical Laboratory Standards Institute; CRP, C-reactive protein; dNTP, deoxynucleotide triphosphates; ESR, erythrocyte sedimentation rate; MIC, minimal inhibitory concentration; MICBR, minimal inhibitory concentration for bacterial regrowth from the biofilm; PBS, phosphate buffered saline; PJI, periprosthetic joint infections; ROC, receiver operating characteristic; TCP, tissue culture plate; WBC, white blood cell.
